# The Prognostic Value of Serum Soluble TREM-1 on Outcome in Adult Patients with Sepsis

**DOI:** 10.3390/diagnostics11111979

**Published:** 2021-10-25

**Authors:** Chia-Te Kung, Chih-Min Su, Sheng-Yuan Hsiao, Fu-Cheng Chen, Yun-Ru Lai, Chih-Cheng Huang, Cheng-Hsien Lu

**Affiliations:** 1 Department of Emergency Medicine, Chang Gung Memorial Hospital-Kaohsiung Medical Center, Chang Gung University College of Medicine, Kaohsiung 833, Taiwan; kungchiate@gmail.com (C.-T.K.); mitosu@gmail.com (C.-M.S.); u9100027@gmail.com (S.-Y.H.); fuchangchen@gmail.com (F.-C.C.); 2 Neurology, Chang Gung Memorial Hospital-Kaohsiung Medical Center, Chang Gung University College of Medicine, Kaohsiung 833, Taiwan; yunrulai@cgmh.org.tw (Y.-R.L.); hjc2828@gmail.com (C.-C.H.); 3 Department of Biological Science, National Sun Yat-sen University, Kaohsiung 833, Taiwan; 4 Center for Shockwave Medicine and Tissue Engineering, Chang Gung Memorial Hospital-Kaohsiung Medical Center, Chang Gung University College of Medicine, Kaohsiung 833, Taiwan; 5 Department of Neurology, Xiamen Chang Gung Memorial Hospital, Xiamen 361000, China

**Keywords:** hospital mortality, sTREM-1, sepsis, outcome

## Abstract

Increased soluble triggering receptor expressed on myeloid cells 1 (sTREM-1) levels have been reported in patients with sepsis. We tested the hypotheses that serum sTREM-1 levels increase in the early phase of sepsis and decrease after sepsis under appropriate treatment and that sTREM-1 levels can predict therapeutic outcomes. One hundred and fifty-five patients prospectively underwent blood samples including biochemical data, sTREM-1, and biomarkers on endothelial dysfunction as well as clinical severity index examinations. Blood samples from Days 1, 4, and 7 after admission were checked. For comparison, 50 healthy subjects were selected as healthy control. Those patients who had sepsis had significantly higher sTREM-1 levels than those of healthy control. sTREM-1 levels positively correlated with biomarkers for endothelial dysfunction (ICAM-1, VCAM-1, and E-selectin) and lactate level as well as clinical severity index (maximum 24 h APACHE score and Sequential Organ Failure Assessment (SOFA) score) upon admission. sTREM-1 concentrations were significantly higher from Day 1 to Day 7 in the non-survivors than in the survivors. A stepwise logistic regression analysis showed only sTREM-1 level and maximum 24 h SOFA score upon admission were significantly associated with fatality. Area under the receiver operating characteristic curve analysis for the diagnostic accuracy of sTREM-1 in sepsis-related fatality gave a value of 0.726, with a cutoff value of 384.6 pg/mL (sensitivity = 80.8% and specificity = 61.5%). sTREM-1 level may be valuable in auxiliary diagnosis, and it can serve as a useful biomarker as a screening service and follow-up therapeutic outcomes in sepsis.

## 1. Introduction

Despite the advent of new antimicrobial drugs and modern equipment, sepsis remains one of the major causes of fatality, with a fatality rate of 20–60% [1]. The pathogenesis of sepsis is characterized by dysregulation of the host response to infection [2], with the entire course having a biphasic process including early pro-inflammatory processes and late-occurring anti-inflammatory processes. Acute infection triggers an overwhelming pro-inflammatory host response, leading to uncoordinated upregulation and downregulation of several different inflammatory pathways. Finally, it results in a dysregulated inflammatory response, and leads to organ dysfunction and failure [3]. In early pro-inflammatory processes of sepsis, biomarkers involved in the pro-inflammatory response of the immune system (e.g., triggering receptor expressed on myeloid cells 1 (TREM-1)), by way of regulating the neutrophil inflammatory responses, have an important effect on the acute host response to sepsis [4,5,6,7]. TREM-1 is expressed on neutrophils and macrophages, regulates and amplifies the secretion of cytokine, and causes granulocyte differentiation and degranulation [8]. An animal study showed a reduction of inflammatory mediators and prolonged survival in mice with infection after blocking of TREM-1 signaling [9]. The value of the sTREM-1 level on assessment of sepsis and its severity has been widely explored [10,11,12,13]. Although clinical studies have investigated the potential role of sTREM-1 on outcome, the results often showed different prognostic effectiveness [13,14,15,16,17,18,19,20,21,22,23]. If blood samples taken from each patient could follow a standard pattern and temporal relationship rather than consisting of only one blood sample on admission, it would reduce variability and improve diagnostic accuracy to predict the prognoses.

According to our hypotheses, sTREM-1 levels are increased in the early phase of sepsis and decrease after sepsis under appropriate treatment, which includes resuscitation strategies and infection control, and sTREM-1 levels can predict therapeutic outcomes. Through this prospectively study, we aimed to study the relationship between serial sTREM-1 levels and therapeutic outcome in patients with sepsis.

## 2. Materials and Methods

### 2.1. Study Design and Patient Selection

This prospective study enrolled 155 nontraumatic patients aged ≥18 years with sepsis in the emergency department (ED) from Kaohsiung Chang Gung Memorial Hospital, one of the most reputed medical centers in southern Taiwan. Diagnostic criteria for sepsis were in accordance with the third international consensus definitions for sepsis and septic shock (Sepsis-3) [2]. Septic shock was defined by a vasopressor agent to maintain a mean arterial pressure of ≥65 mm Hg and serum lactate level of ≥2 mmol/L (>18 mg/dL) without the condition of hypovolemia. Inclusion criteria included patients with sepsis as well as a Sequential Organ Failure Assessment (SOFA) score of ≥2 points. Exclusion criteria included (1) patients who had comorbidities that could affect the results (e.g., malignance that underwent chemotherapy) and (2) families signed a do-not-resuscitate order upon ED admission. In this study, 50 healthy volunteers without evidence of infection were enrolled as the healthy control. The Institutional Review Committees on Human Research in our hospital approved the study (protocol code: 103-5216B and date of approval 23 October 2014). All participants (patients or families) signed informed consent.

### 2.2. Clinical Protocol

Medical records were collected using standardized evaluation forms including the infection origin, administration of antimicrobial agents, various organ dysfunctions, vasoactive agent supplementation, and ventilator support or not. The clinical scale for disease severity and organ malfunction including Acute Physiology and Chronic Health Evaluation (APACHE) II score, and the SOFA score to identify organ failure, was evaluated within the first 24 h in the emergency department (ED). In our institutional, an infectious disease expert is routinely consulted to choose an appropriate antimicrobial regimen.

### 2.3. Collection and Processing of Basic Laboratory Tests

Biochemical laboratory tests, lactate concentration, and biomarkers for infection, including C-reactive protein (CRP) and procalcitonin, were collected in accordance with well-established methods by our hospital’s central laboratory [24,25,26].

### 2.4. Assay for the sTREM-1 and Biomarkers for Endothelial Dysfunction

After enrollment, blood samples of serum sTREM-1 and biomarkers for endothelial dysfunction, including ICAM-1, VCAM-1, E-selectin, and P-selectin, were collected on Day 1 as well as on Day 4 and Day 7. All serum samples were collected after centrifugation, isolated, and stored at −80 °C in multiple aliquots. Biomarkers were assayed using commercially available ELISA (R & D Systems, Minneapolis, MN, USA). The detailed methodology was described in a previously study [26].

### 2.5. Outcome Analysis

Patients were classified into survivors and non-survivors in accordance with 28-day in-hospital survival status. Furthermore, we evaluated the prediction ability of TREM-1 (Day 1) in organ dysfunction presented in the first 48 h of admission. Acute kidney injury (AKI) was defined according to KDIGO guidelines [27]. Intubation and ventilator usage after sepsis-related respiratory failure was also recorded.

### 2.6. Statistical Analysis

Continuous variables were expressed as the mean ± standard deviation (SD). Pearson correlation analysis was to evaluate the relationships between clinical severity scales and the biomarkers in patients with sepsis upon admission. Repeated measurements of analysis of variance (ANOVA) was used to compare the serial changes of sTREM-1 between survivors and non-survivors at 3 different periods (Days 1, 4, and 7). Stepwise logistic regression was used to explore the significant variables on therapeutic outcomes. Receiver operating characteristic (ROC) curves were used to evaluate the diagnostic accuracy for potential risk factors for sepsis-related fatality. All these analyses were performed using SPSS for Windows, version 18.

## 3. Results

### 3.1. Characteristics of Patients and Healthy Control

There were 155 patients and 50 healthy controls enrolled in the present study (Table 1). The mean age of patients was 64.5 (SD, 13.8) years, and the 28-day fatality rate was 18.7% (29/155). Demographic data including age, sex, and underlying diseases did not show statistically significant differences between the two groups. Both WBC counts and CRP levels were higher in the sepsis group, while platelet and Hb levels were lower in the sepsis group. Moreover, serum sTREM-1 levels were significantly higher in the sepsis group than in the healthy control group (496.5 ± 382.3 vs. 60.4 ± 44.8; *p* < 0.001).

### 3.2. Correlation Analysis between sTREM-1 and Biomarkers for Endothelial Dysfunction, and Clinical Severity Index

**Figure 1:**
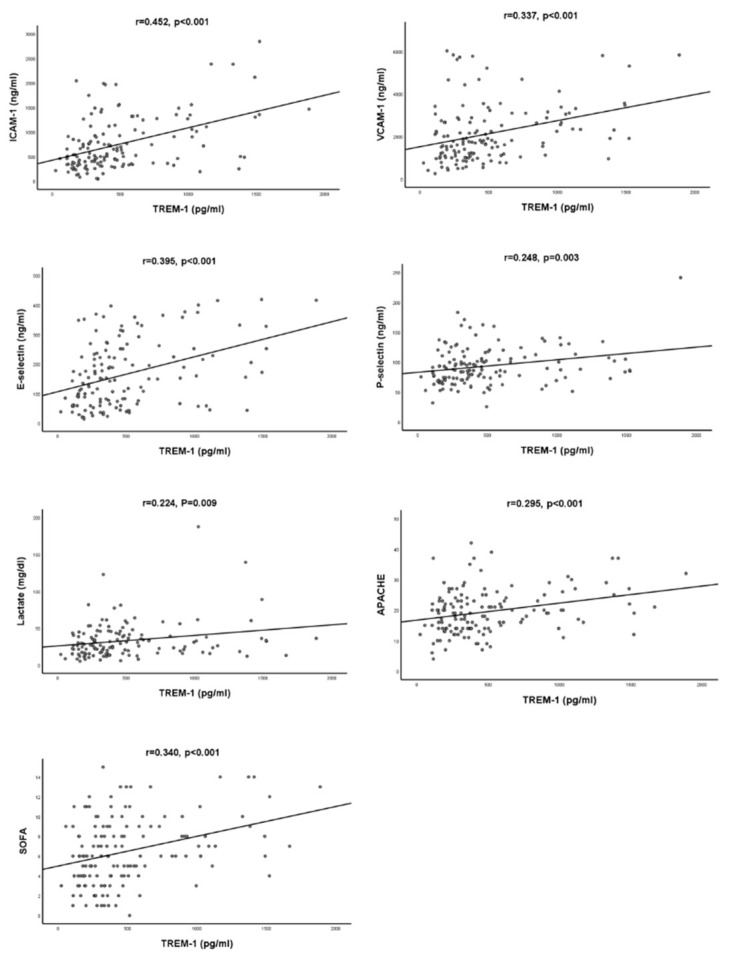
Scatterplot of serum sTREM-1 with other biomarkers and clinical sores.

The correlation analysis was explored to investigate the relationship between biomarkers, including sTREM-1 and biomarkers for endothelial dysfunction, and clinical severity indexes and is presented in Table 2 and Figure 1. sTREM-1 level was significantly associated with ICAM-1 (r = 0.452, *p* < 0.001), VCAM-1 (r = 0.337, *p* < 0.001), E-selectin (r = 0.395, *p* < 0.001), P-selectin (r = 0.248, *p* = 0.003), lactate (r = 0.224, *p* = 0.009), maximum 24 h SOFA score (r = 0.340, *p* < 0.001), and the maximum 24 h APACHE score (r = 0.295, *p* < 0.001) upon admission.

### 3.3. Comparison of the Characteristics between Survivors and Non-Survivors

Clinical characteristics and laboratory data between the survival and non-survival groups are listed in Table 3. There were no statistically significant differences in age, sex, and underlying diseases between the two groups. The most common microorganisms were *Escherichia coli* (14.2%) in survival and *Klebsiella pneumoniae* (13.8%) in non-survival. The most common infection focus was urinary tract infection in survival and respiratory tract infection in non-survival. Those patients who were non-survivors had a significant higher respiratory failure rate within 24 h compared with the survivors (69.0% vs. 28.9%, *p* < 0.001). There were 100 patients who suffered from septic shock within 24 h after admission, accounting for 64.5% of patients, but there were no statistical differences between survivors and non-survivors (61.1% vs. 79.3%). Those patients who were non-survivors also had a significantly higher maximum 24 h APACHE II score and maximum 24 h SOFA score than those of the survivors (22.8 ± 8.3 vs. 18.5 ± 6.9, *p* = 0.004; 8.4 ± 3.5 vs. 5.9 ± 3.2, *p* < 0.001, respectively). Moreover, significantly higher lactate, sTREM-1, ICAM-1, and VCAM-1 levels and lower platelet counts were observed in non-survivors than in the survivors (44.6 ± 35.7 vs. 30.7 ± 23.2, *p* = 0.012; 732.4 ± 441.7 vs. 444.1 ± 348.8, *p* < 0.001; 1010.6 ± 638.2 vs. 701.0 ± 526.8, *p* = 0.02; 2746.7 ± 1517.0 vs. 1951.7 ± 1263.7, *p* = 0.004; 150.5 ± 74.5 vs. 189.7 ± 99.9, *p* = 0.049, respectively). However, there were no statistical differences in E-selectin and P-selectin levels between the two groups.

### 3.4. Serial Changes of Serum sTREM-1 Level between Survivor and Non-Survivors

**Figure 2:**
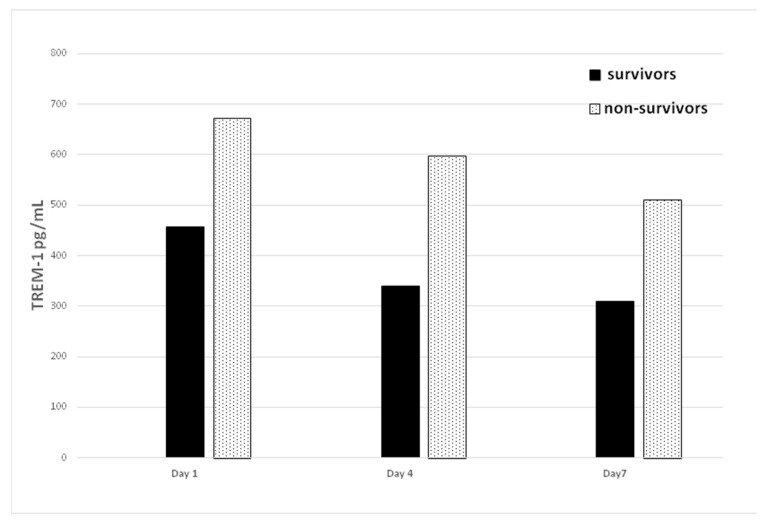
Comparison of serum sTREM-1 level between survivors and non-survivors on different days.

Serial sTREM-1 levels were monitored in patients with sepsis between survivors and non-survivors on Days 1, 4, and 7 and are presented in Figure 2. sTREM-1 levels initially increase during sepsis and decrease after sepsis under appropriate control. Non-survivors showed significantly higher sTREM-1 concentrations than did survivors on Day 1, Day 4, and Day 7. Additionally, repeated measurements ANOVA demonstrated that sTREM-1 levels between survivors and non-survivors at three different periods (Day 1, 4, and 7) were significantly different (*p* = 0.018).

### 3.5. Prognostic Value of Biomarkers and Clinical Severity Scores on Outcome

**Figure 3:**
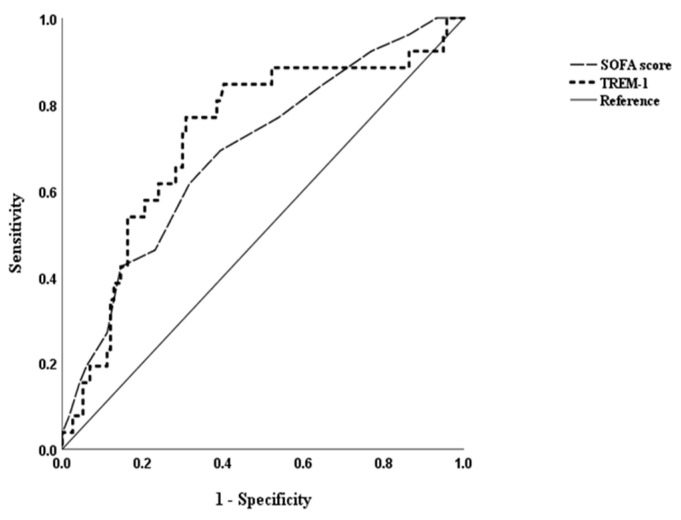
ROC curve of sTREM-1 in predicting sepsis related mortality.

The baseline variables on Day 1 showed several significantly different variables between the two groups (survivors and non-survivors), including lactate, sTREM-1, ICAM-1, and VCAM-1, APACHE II, and SOFA scores on univariate analysis. These statistically significant variables were put into a stepwise logistic regression analysis model, and the results showed that only sTREM-1(*p* = 0.028) and maximum 24 h SOFA score (*p* = 0.042) upon admission were independently associated with sepsis-related fatality (Table 4). Any increase of 1 pg/mL in sTREM-1 will increase the fatality rate by 0.1%. The AUCs for sTREM-1 and SOFA score were 0.726 (95% CI: 0.613–0.838) and 0.705 (95% CI: 0.602–0.808), respectively (Figure 3). The cutoff values for diagnostic accuracy in presence of fatality were 384.6 pg/mL (sensitivity = 80.8% and specificity = 61.5%) and 6.5 (sensitivity = 69% and specificity = 63.5%), respectively.

We also evaluated the predictive ability of 28-days mortality by the drop percentage of sTREM-1 between Day 1 to Day 4 and Day 1 to Day 7 with the ROC curves analysis. The AUCs of sTREM-1 were only 0.403 (95% C.I. 0.242~ 0.564, *p* = 0.207) for Day 1 to Day 4 and 0.559 (95% C.I. 0.395~0.723, *p* = 0.469) for Day 1 to Day 7. The results were poor and were not better then single sTREM-1 data.

### 3.6. Predictive Ability of sTREM-1 for Septic Shock, Organ Dysfunction, and Bacteremia

The potential predictive ability of sTREM-1 for septic shock, acute kidney injury, mechanical ventilation, and bacteremia in patients with sepsis is listed in Table 5. sTREM-1 was significantly higher in the bacteremia group than in the nonbacteremia patients (*p* = 0.023). There was no significant difference in sTREM-1 levels between those in the AKI group and those in the non-AKI group. There were also no statistical differences in predicting septic shock and respiratory failure between the two groups.

## 4. Discussion

### 4.1. Major Findings

Our study investigated serial sTREM-1 levels during the acute phase of sepsis and confirmed the hypotheses that sTREM-1 levels increase in the early phase of sepsis and decrease after antimicrobial therapy and that sTREM-1 levels on admission can predict treatment outcomes. In addition, the sTREM-1 level is positively correlated with biomarkers for endothelial dysfunction (ICAM-1, VCAM-1, and E-selectin) and lactate level, clinical severity index (the maximum 24 h APACHE score and maximum 24 h SOFA score) upon admission. Further, sTREM-1 could provide the diagnostic accuracy for sepsis-related fatality (AUC = 0.726) with a cutoff value of 384.6 pg/mL (sensitivity = 80.8% and specificity = 61.5%). Any increase of 1 pg/mL in the sTREM-1 level would increase the fatality rate by 0.1%.

### 4.2. The Role of sTREM-1 Levels on Fatality

Several biomarkers (e.g., CRP, procalcitonin, WBC and differential counts, lactate, and interleukin-6) had already been investigated for their role of sepsis-related fatality [28,29]. Studies showed that sTREM-1 level was higher in the non-survivor than in the survivor during the early phase of sepsis [18,19], which is consistent with our study. Another study demonstrated that sTREM-1 can predict the 28-day sepsis-related fatality but its level did not correlate with clinical severity scores [15]. In contrast, our study also showed that the sTREM-1 level is positively correlated with clinical severity index (the maximum 24 h APACHE score and maximum 24 h SOFA score) upon admission. Another study showed that it is not sTREM-1 level at early stage on admission but progressive increases in serum sTREM-1 levels during hospitalization that are associated with an excess of fatality in patients with sepsis [20,21]. On the other hand, several studies found that sTREM-1 level was significantly lower in the non-survivors than in the survivors during the acute phase of sepsis [13,14]. Finally, one meta-analysis demonstrated that an elevated sTREM-1 level was associated with a higher risk of death in infection, with a relative risk of 2.54 [30].

### 4.3. Possible Mechanism in This Study

There are several possible reasons to explain our observation. First, a higher sTREM-1 level on admission implies more severe cell and tissue damage by proteinases released from pathogens or necrotic cells, which contributes to a higher fatality rate. Second, sTREM-1 may have an anti-inflammatory effect [19]. The organs are overwhelmed by massive amounts of bacteria-produced endotoxins (e.g., lipopolysaccharide) in the late-occurring anti-inflammatory stage. The persistent excessive inflammatory response contributes to both continuous high expressions of membrane-bound TREM-1 and increased sTREM-1 levels. Third, sTREM-1 may also act as a pro-inflammatory mediator that produces an increased expression of endothelial dysfunction via a higher inflammatory state. Clinical study demonstrated that sTREM-1 acts in the trans-epithelial migration process of leukocytes and more specifically of monocytes through expression of selectin [31]. Another study supports the importance of sTREM-1 in sepsis, showing that the trans-epithelial migration of neutrophils and monocytes is increasing in sepsis and migrating neutrophils bind to sTREM-1 [32].

Higher sTREM-1 levels are associated with endothelial dysfunction. Endothelial dysfunction reflects vasodilatation, hypotension with decreased perfusion, and eventually multiple organ dysfunction [33]. Our study showed that sTREM-1 concentration highly correlated with biomarkers for endothelium dysfunction (ICAM-1 and VCAM-1). These biomarkers are related to trans-endothelial migration of activated leukocytes to the endothelium and subsequent microvascular injury, subsequently leading to multiple organ failures during sepsis [33]. This increased expression of endothelial dysfunction contributes to worse outcomes in the non-survivors. Lastly, a previous study showed that serum sTREM-1 levels correlated with disease severity and independent risk factors associated with myocardial dysfunction in patients with sepsis [34].

In our study, we also found SOFA score and sTREM-1 levels upon admission were both significantly associated with sepsis-related fatality. SOFA score is based on six different scores, which included respiratory, cardiovascular, hepatic, coagulation, renal, and neurological systems. Therefore, the clinical use of SOFA score is more complicated than that of the sTREM-1 level. Although lactate was also a significant predictor on outcome, both sTREM-1 and SOFA scores were superior to lactate for outcome prediction in this study.

## 5. Study Limitations

The strength of this study had several limitations. First, the therapeutic strategy for sepsis (e.g., use of steroids or not, and antimicrobial agents) may be different based on the preference of the doctor. This may exist as a potential bias in the statistical analysis. Second, some of our patients enrolled in this study before the new sepsis definition had been revised, therefore, we reclassified these patients according to the new sepsis definition.

## 6. Conclusions

Our study confirmed that sTREM-1 levels increase in the early phase of sepsis and decrease after sepsis under appropriate control and that sTREM-1 levels on admission can predict therapeutic outcomes. The sTREM-1 level may be valuable in auxiliary diagnosis, and it can serve as a useful biomarker as a screening service and follow-up therapeutic outcome in sepsis.

## Figures and Tables

**Table 1:** Baseline characteristics of patients and control subjects.

	Control	Study Patients	*p-*Value
	*n* = 50	*n* = 155	
Age (mean ± SD)	56.4 ± 12.0	64.5 ± 13.8	NS
Gender (male%)	52.9	66.5	NS
Underlying disease (%)			
Diabetes mellitus	33.4	37.4	NS
Hypertension	46.5	49.7	NS
Coronary artery disease	8.7	9.0	NS
Laboratory data (mean ± SD)			
White blood cell (10^9^/L)	5.6 ± 1.6	16.1 ± 10.6	<0.001 *
Platelet (10^4^/L)	223.7 ± 61.9	182.3 ± 96.6	<0.001 *
Hemoglobin (mg/dL)	14.3 ± 1.9	11.8 ± 2.4	<0.001 *
C-reactive protein (mg/L)	1.4 ± 1.3	191.3 ± 117.6	<0.001 *
sTREM-1 (pg/mL)	60.4 ± 44.8	496.5 ± 382.3	<0.001 *

Abbreviation: SD, standard deviation; NS, not significant; sTREM-1, soluble triggering receptor expressed on myeloid cells-1, * *p* < 0.05.

**Table 2:** Correlation analysis between sTREM-1 and biomarkers for endothelium dysfunction and clinical severity index.

	sTREM-1	
	r	*p* Value
ICAM-1	0.452	<0.001 *
VCAM-1	0.337	<0.001 *
E-selectin	0.395	<0.001 *
P-selectin	0.248	0.003 *
lactate	0.224	0.009 *
APACHE	0.295	<0.001 *
SOFA	0.340	<0.001 *

Abbreviation: ICAM-1, intercellular adhesion molecule-1; VCAM-1, vascular cell adhesion molecule-1; APACHE, Acute Physiology and Chronic Health Evaluation; SOFA, Sequential Organ Failure Assessment; sTREM-1, soluble triggering receptor expressed on myeloid cells-1, * *p* < 0.05.

**Table 3:** Characteristics of survivors and non-survivors of septic patients.

	Survivors (*n* = 126)	Non-Survivors (*n* = 29)	*p-*Value
Age (mean ± SD)	64.0 ± 13.9	67.3 ± 13.6	NS
Female/Male	44/82	8/21	NS
Underlying disease (%)			
Diabetes mellitus	44(34.9%)	14(48.3%)	NS
Hypertension	64(50.8%)	13(44.8%)	NS
Liver cirrhosis	16(12.7%)	6(20.7%)	NS
Chronic lung disease	17(13.5%)	4(13.8%)	NS
Stroke	21(16.7%)	6(20.7%)	NS
Coronary artery disease	12(9.5%)	2(6.9%)	NS
Chronic renal disease	28(22.2%)	8(27.6%)	NS
Solid tumor	19(15.1%)	9(31.0%)	NS
Clinical presentation (mean ± SD) ^a^			
Systolic blood pressure	110.2 ± 41.8	108.3 ± 39.8	NS
Pulse rate	108.4 ± 24.5	112.8 ± 30.0	NS
Shock within 24 h	77(61.1%)	23(79.3%)	NS
Respiratory failure within 24 h	36(28.9%)	20(69.0%)	<0.001 *
Disease severity index (mean ± SD)			
Maximum 24 h APACHE II score	18.5 ± 6.9	22.8 ± 8.3	0.004 *
Maximum 24 h SOFA score	5.9 ± 3.2	8.4 ± 3.5	<0.001 *
Bacteremia	41(32.5%)	10(34.5%)	NS
Steroids use	36 (28.6%)	11 (37.9%)	NS
Inotropic agents use	42 (33.3%)	12 (41.3%)	NS
Inappropiate initial antibiotics	12 (9.5%)	2 (6.9%)	NS
Laboratory data (mean ± SD) ^a^			
White blood cell count (10^9^/L)	15.2 ± 9.9	19.8 ± 12.8	NS
Neutrophil (%)	80.8 ± 14.9	79.2 ± 17.1	NS
Hemoglobin (mg/dL)	11.9 ± 2.2	11.6 ± 3.0	NS
Platelet counts (10^4^/L)	189.7 ± 99.9	150.5 ± 74.5	0.050
C-reactive protein (mg/L)	195.5 ± 119.5	172.9 ± 109.1	NS
Lactate (mg/dL)	30.7 ± 23.2	44.6 ± 35.7	0.012 *
Creatinine (mg/dL)	2.35 ± 2.18	3.03 ± 3.73	NS
T-bilirubin (mg/dL)	1.86 ± 2.40	2.85 ± 3.50	NS
sTREM-1 (pg/mL)	444.1 ± 348.8	732.4 ± 441.7	<0.001 *
ICAM-1 (ng/mL)	701.0 ± 526.8	1010.6 ± 638.2	0.020 *
VACM-1 (ng/mL)	1951.7 ± 1263.7	2746.7 ± 1517.0	0.004 *
E-selectin (ng/mL)	158.7 ± 110.0	200.0 ± 124.4	NS
P-selectin (ng/mL)	92.2 ± 28.8	101.0 ± 44.2	NS

Abbreviation: SD, standard deviation; APACHE, Acute Physiology and Chronic Health Evaluation; SOFA, Sequential Organ Failure Assessment; sTREM-1, soluble triggering receptor expressed on myeloid cells-1; ICAM-1, intercellular adhesion molecule-1; VCAM-1, vascular cell adhesion molecule-1; NS, not significant, * *p* < 0.05. ^a^ The values of BP, heart rate and nontarget laboratory parameters were obtained at the triage and first time of evaluation.

**Table 4:** ROC curve analysis of different biomarkers or score in predicting sepsis-related fatality.

	AUC	(95% Confidence Interval)	Cut off Point	Sensitivity	Specificity
TREM-1	0.726 *	0.613~0.838	384.6 pg/mL	0.808	0.615
ICAM-1	0.635 *	0.510~0.759	597 ng/mL	0.615	0.550
VCAM-1	0.655 *	0.535~0.774	1960 ng/mL	0.654	0.631
Lactate	0.644 *	0.535~0.752	2 mg/dL	0.857	0.325
SOFA	0.705 *	0.602~0.808	6.5	0.690	0.635
APACHE	0.658 *	0.548~0.769	17.5	0.793	0.492

Abbreviation: sTREM-1, soluble triggering receptor expressed on myeloid cells-1; ICAM-1, intercellular adhesion molecule-1; VCAM-1, vascular cell adhesion molecule-1; APACHE, Acute Physiology and Chronic Health Evaluation; SOFA, Sequential Organ Failure Assessment, * *p* < 0.05.

**Table 5:** sTREM-1 in predicting different organ dysfunction.

	Case Numbers (*n*)	TREM-1 Level			AUC	95% Confidence Interval
		Present	Absent	*p* Value		
Bacteremia	51	613.9 ± 464.3	439.1 ± 332.4	0.023 *	0.593	0.491~0.696
AKI	59	557.7 ± 362.7	460.5 ± 390.9	NS	0.620 *	0.526~0.714
Septic shock	100	536.8 ± 401.4	419.2 ± 333.0	NS	0.587	0.491~0.682
Respiratory failure	56	552.5 ± 411.7	463.5 ± 362.2	NS	0.567	0.468~0.666

Abbreviation: sTREM-1, soluble triggering receptor expressed on myeloid cells-1; AKI, acute kidney injury; AUC, area under curve; NS: not significant; * *p* < 0.05.

## Data Availability

The data presented in this study are available on request from the corresponding author. The data are not publicly available due to their containing information that could compromise the privacy of research participants.

## Abbreviations

TREM-1: triggering receptor expressed on myeloid cells 1; sTREM-1: soluble triggering receptor expressed on myeloid cells 1; ICAM1: intercellular adhesion molecule 1; VCAM1: vascular cell adhesion molecule 1; APACHE: Acute Physiology and Chronic Health Evaluation; SOFA: Sequential Organ Failure Assessment; ED: emergency department; CRP: C-reactive protein; AKI: acute kidney injury; KDIGO: Kidney Disease: Improving Global Outcomes; sCr: serum creatinine.
